# Genomic and Functional Characterization of Poultry *Escherichia coli* From India Revealed Diverse Extended-Spectrum β-Lactamase-Producing Lineages With Shared Virulence Profiles

**DOI:** 10.3389/fmicb.2019.02766

**Published:** 2019-12-03

**Authors:** Arif Hussain, Sabiha Shaik, Amit Ranjan, Arya Suresh, Nishat Sarker, Torsten Semmler, Lothar H. Wieler, Munirul Alam, Haruo Watanabe, Dipshikha Chakravortty, Niyaz Ahmed

**Affiliations:** ^1^Department of Microbiology and Cell Biology, Indian Institute of Science, Bangalore, India; ^2^International Centre for Diarrhoeal Disease Research, Bangladesh (icddr,b), Dhaka, Bangladesh; ^3^Pathogen Biology Laboratory, Department of Biotechnology and Bioinformatics, University of Hyderabad, Hyderabad, India; ^4^Methodology and Research Infrastructure, Robert Koch Institute, Berlin, Germany; ^5^International University of Health and Welfare, Tokyo, Japan

**Keywords:** poultry *Escherichia coli*, genomics, ESBL, ExPEC, India

## Abstract

Extended-spectrum β-lactamases (ESBLs) form the most important resistance determinants prevalent worldwide. Data on ESBL-producing *Escherichia coli* from poultry and livestock are scarce in India. We present data on the functional and genomic characterization of ESBL-producing *E. coli* obtained from poultry in India. The whole genome sequences of 28 ESBL-producing *E. coli* were analyzed comprising of 12 broiler chicken *E. coli* isolates, 11 free-range chicken *E. coli* isolates, and 5 human extraintestinal pathogenic *E. coli*. All of the 28 ESBL-producing *E. coli* isolates were tested for antibiotic susceptibilities, *in vitro* conjugation, and virulence-associated phenotypic characteristics. A total of 13 sequence types were identified from the poultry *E. coli*, which included globally successful sequence types such as ST117 (9%), ST131 (4.3%), and ST10 (4.3%). The most common ESBL gene detected in poultry *E. coli* genomes was *bla*_CTX-M-15_ (17%). Also, FIB (73%) and FII (73%) were the most common plasmid replicons identified. Conjugation experiments demonstrated 54 (7/13), 30 (3/10), and 40% (2/5) of broiler, free-range, and human ExPEC *E. coli* to be able to transfer their ESBL genes, respectively. The *in vitro* virulence-associated phenotypic tests revealed the broiler, free-range, and human ExPEC isolates to be comparable in biofilm formation, resistance to serum bactericidal activity, adherence, and invasion capabilities. Our overall results showed prevalence of virulence phenotypes among the diverse ESBL-producing *E. coli* from poultry; while certain *E. coli* clones from broiler-poultry may indeed have the potential to cause infection in humans.

## Introduction

Extended-spectrum-β-lactamases (ESBLs)-producing *Enterobacteriaceae* have become a serious problem worldwide adversely impacting the treatment of infectious diseases (Miyoshi-Akiyama et al., 2016). Infections with ESBL-producing *Escherichia coli* are associated with a range of conditions resulting in increased morbidity, mortality, and healthcare costs (Tumbarello et al., 2010). The prevalence of *E. coli* resistant to extended-spectrum-β-lactam antibiotics is highest among developing countries (Byarugaba, 2004). In India, high levels of cephalosporin resistance have been reported (16–95%) in *E. coli* following extensive use of ceftriaxone (Miyoshi-Akiyama et al., 2016). ESBLs are beta-lactamases that confer resistance to penicillins, third-generation cephalosporins, and monobactams (Rawat and Nair, 2010). Restricted treatment options due to the emergence of multi-drug resistant phenotypes in ESBL-producing bacteria have led to increased dependence on and subsequent increase in the prevalence of *E. coli* resistant to last-line antibiotics such as carbapenems (Ranjan et al., 2016).

Although ESBL production was initially reported in hospital-acquired infections caused mainly by *Klebsiella pneumoniae*, currently, its associations with community-acquired infections, particularly with urinary tract infections caused by *E. coli* have been frequently reported (Paterson and Bonomo, 2005). The commensal *E. coli* in the fecal carriage are thought to be one of the main reservoirs of ESBL-*E. coli* in the community settings (Kluytmans et al., 2013). Scenarios involving transmission of ESBL-producing *E. coli* in food animals and humans have been reported in several studies (Kluytmans et al., 2013; Mitchell et al., 2015; Robinson et al., 2016). We recently reported ESBL-producing *E. coli* in retail chicken meat in India, wherein close resemblance of genotypes was also identified between *E. coli* from chicken meat and human ExPEC pathotypes (Hussain et al., 2017). Several studies have suggested that *E. coli* from poultry represents significant zoonotic potential (Kluytmans et al., 2013; Mitchell et al., 2015; Robinson et al., 2016; Hussain et al., 2017). Molecular determinants of ESBLs are frequently found on plasmids that could also harbor virulence genes and these can be horizontally transferred to other bacteria, even to bacteria of other genera (Skyberg et al., 2006; Hussain et al., 2012). Presence of virulence factors in ESBL-producing *E. coli* is an important factor in the epidemiology of drug-resistant infections (Johnson et al., 2006; Jadhav et al., 2011), as exposure to ESBL-producing pathogenic *E. coli* strains may cause hard-to-treat infections, also in healthy individuals.

Worldwide, significantly large geographic differences are observed in the prevalence of ESBL genes. However, *bla*_CTX-M_ types and in particular, the *bla*_CTX-M-15_ are now the predominant and the most extensively disseminated ESBL genotypes found among *E. coli* isolates (Hussain et al., 2014; Ranjan et al., 2015, 2017). The ESBL-producing *E. coli* are also predominantly linked with globally disseminating ST131 clones and are frequently associated with fluoroquinolone resistance (Hussain et al., 2014; Nandanwar et al., 2016; Shaik et al., 2017). Whole genome sequencing (WGS) is an important tool in analyzing the emergence and spread of antimicrobial resistant isolates, and therefore, may help in formulating strategies in controlling antimicrobial resistance.

Although studies have investigated the epidemiology of ESBL-producing *E. coli* of poultry origins, which may be directly linked to public health (Mellata, 2013; Mitchell et al., 2015; Hussain et al., 2017), the aim of this study is to further obtain a comprehensive understanding of the differences and or similarities in genomic and functional attributes of ESBL-producing *E. coli* obtained from free-range (country chicken) and farm-raised (broiler) poultry in India by integrating new data regarding phenotypic virulence traits, extensive antimicrobial resistance screening, whole genome sequencing, and comparative genomics. In this study, we identified prevalence of virulent phenotypes among diverse ESBL-*E. coli* lineages from poultry. Particularly, the whole genome sequencing revealed that the broiler *E. coli* isolates shared affinities with human ExPEC isolates with regard to virulence, resistance, and phylogenetic backgrounds.

## Materials and Methods

### Strain Collection

A total of 28 ESBL-producing *E. coli* isolates were analyzed in this study that included; 23 ESBL *E. coli* isolates that were randomly selected comprising of 11 free-range *E. coli* and 12 broiler *E. coli*, which were a part of an earlier described collection of 168 poultry *E. coli* from retail chicken meat and carcass isolated from three cities representing three states in India during February 2015 – September 2015 (Hussain et al., 2017). The remaining five ESBL-producing-*E. coli* used in this study were human ExPEC isolates, which were part of the previously described reports from India (Avasthi et al., 2011; Ranjan et al., 2016; Shaik et al., 2017). Details of all the strains employed in this study are tabulated in Supplementary Table S1. Broiler chicken represents farm-raised broiler chicken category that was fed on commercial feed and raised for meat production; the free-range chicken represents the country (native) birds that were reared in backyard farms and households (not raised in farms).

### Whole Genome Sequencing, Phylogenomic Analysis, and Genetic Characterization

Genomic DNA from 13 strains (randomly selected ESBL-*E. coli* respresenting three geographical locations: Karnataka, Andhra Pradesh and Telangana) was isolated using the Qiagen DNeasy blood and tissue kit (Qiagen, Germany) (Qumar et al., 2017). Illumina MiSeq reads of 13 strains (Supplementary Table S1) were filtered for high quality reads using Trimmomatic (v0.36) (Bolger et al., 2014) and NGS QC Toolkit (v2.3.3) (Patel et al., 2012) and the reads of three Free-range *E. coli* strains (NAEC1123, NAEC1145, and NAEC1148) were excluded due to poor quality. The high quality reads of 10 strains were then assembled *de novo* using SPAdes Genome Assembler (v3.6.1) (Bankevich et al., 2012) and further ordered and scaffolded using Contig-Layout-Authenticator (Shaik et al., 2016). The ordered scaffolds were then annotated using RAST server (Overbeek et al., 2014) and the statistics were gleaned using ARTEMIS (Rutherford et al., 2000). The genomic features of 10 poultry *E. coli* genomes are represented in Supplementary Table S3.

In addition to these newly sequenced 10 genomes (8 Free-range and 2 Broiler), previously studied 10 ESBL *E. coli* genomes from broiler and 5 genomes from human hosts (with an inclusion criteria of >4,500 CDS and above 80% coding percentage) were also included for the entire analysis (Supplementary Table S1). The sequence type (ST) and plasmid profiles of all the 25 strains were determined *in silico*.^1^ Phylogroups of *E. coli* genomes were determined by an *in silico* Clermont typing method (Beghain et al., 2018). A Harvest (Treangen et al., 2014) based core-genome phylogenetic tree of 25 *E. coli* strains was generated by filtering the possible recombining regions, poor alignments, repetitive regions, and the resulting tree was visualized using iTOL (Letunic and Bork, 2016). Virulence and resistance gene screening of these 25 strains was carried out by BLASTp (70% identity and 90% query coverage) analysis against homologous genes (not entire ORFs) present in the Virulence Factors Database (VFDB) and Comprehensive Antibiotic Resistance Database (CARD), respectively. Heatplots depicting the hierarchical clustering were executed in the gplot package of R as described by our group, previously (Ranjan et al., 2016).

### Biofilm Formation

Biofilm production was analyzed in flat-bottom 96-well microtiter plates. The overnight LB culture was diluted to optical density 0.05 (OD 600 nm) in fresh M63 minimal medium, and 200 μl of this dilution was added into the wells in triplicates and covered by a breathable sealing and incubated for 48 h at 28°C in a stationary condition. Biofilms were quantified after 48 h of incubation using crystal violet staining, the optical density of the well containing only bacterium-free medium was used as a control. The absorbance was measured at 570 nm. All tests were carried out at least three times, and the results were averaged. Specific biofilm formation was computed using formula specific biofilm formation (SBF) = (AB − CW)/*G*, where AB = OD 570 nm of the attached and stained bacteria, CW = OD 570 nm of the stained control wells containing only bacteria-free medium, and *G* = OD 600 nm of bacterial growth in broth immediately after 48 h incubation (Hussain et al., 2014).

### Serum Resistance, Adhesion, and Invasion Assays

For serum assay, overnight grown bacterial cultures (5 μl) were added to LB broth (495 μl) and incubated for 1 h in a shaker incubator. These bacterial cultures were then centrifuged, and the pellets were suspended in 1 ml of 1X PBS. About 30 μl of this bacterial suspension was added to 270 μl of 50% (diluted in PBS) human serum (Pan Biotech, Germany) in triplicates in a 96-well microtiter plate. Initially, samples were collected at 0 h, serially diluted in PBS, and plated on LB agar plates for enumerating the colony forming units. The microtiter plate was then incubated for 3 h at 37°C at 100 rpm. After 3 h, samples from each well were aliquoted for serial dilution and plated on LB agar plates. Strains which had equaled or higher colony forming units (CFU) at 3 h compared to 0 h were considered resistant to human serum. The experiment was repeated twice in three technical replicates (Ranjan et al., 2016; Suresh et al., 2018).

Adhesion and invasion assays were performed on T24, human bladder epithelial cells as previously reported (Ranjan et al., 2016). In brief, human bladder epithelial cells were grown in RPMI-1640 in 24 well-plates 48 h prior to cell infection. Following 3 h of infection at 37°C with MOI of 10, the monolayer was washed thrice (using 1X PBS) and lysed (using 0.1% Triton X-100 in 1X PBS). The lysates were serially diluted and plated on LB agar. For the invasion assay, the cells were incubated additionally for 1.5 h with replaced media containing 100 μg/ml gentamicin. The cells were then processed as described for the adhesion assay.

### Antimicrobial Susceptibility and Conjugation Assay

Antimicrobial susceptibility of 28 strains (23 poultry and 5 ExPEC strains) against 11 different antibiotics was carried out on Mueller-Hinton agar (MHA) medium using Kirby-Bauer disc diffusion employing following antibiotic discs: colistin (10 μg), tetracycline (30 μg), chloramphenicol (30 μg), oxytetracycline (30 μg), nalidixic acid (30 μg), streptomycin (10 μg), fosfomycin (50 μg), chlortetracycline (30 μg), cotrimoxazole (25 μg), gentamicin (120 μg), and ciprofloxacin (5 μg). All isolates were tested for MBL production employing imipenem with and without EDTA Ezy MIC Strips (HiMedia, India). Results were interpreted as per the criteria of the Clinical and Laboratory Standards Institute (CLSI) (Ranjan et al., 2016). Conjugative transfer of ESBL genes among ESBL producing poultry *E. coli* was analyzed by *in vitro* conjugation as previously described by our study group, transconjugants were selected on EMB agar containing 100 μg/ml sodium azide and 4 μg/ml cefotaxime (Hussain et al., 2014).

### Statistical Analysis

Statistical analysis for aggregate resistance and virulence gene scores (sum of all resistance/virulence genes for which the isolates were tested positive, respectively) biofilm formation, serum resistance assay, adhesion, and invasion assays were done using the non-parametric Mann-Whitney U-test executed in GraphPad Prism.

### Accession Number(s)

The GenBank accession numbers of the 10 genomes sequenced in this study are RDBX00000000 (NAEC1028), RDBY00000000 (NAEC1042), RDBZ00000000 (NAEC1115), RDCA00000000 (NAEC1132), RDCB00000000 (NAEC1134), RDCC00000000 (NAEC1135), RDCD00000000 (NAEC1136), RDCE00000000 (NAEC1146), RDCF00000000 (NAEC1147), and RDCG00000000 (NAEC1154).

## Results

### Bacterial Isolates

In total, 28 *E. coli* isolates were analyzed in this study that comprised of 5 human ExPEC, 12 broiler *E. coli,* and 11 free-range *E. coli* isolates. All five human ExPEC isolates were originated from urine samples of symptomatic UTI patients, they were collected from Pune city, Maharashtra. Broiler *E. coli* isolates includes 10 *E. coli* obtained from cecum (representing 6 isolates from Telangana, 2 from Karnataka, and 2 from Andhra Pradesh) and 2 from chicken meat (from Telangana). Free-range *E. coli* isolates include six isolates from cecum (representing 4 from Karnataka and 2 from Andhra Pradesh) and five from chicken meat (representing 3 from Telangana, 1 from Karnataka, and 1 from Andhra Pradesh). All the 28 *E. coli* isolates were tested for the *in vitro* associated virulence tests. Out of these, only 25 *E. coli* isolates were used for genomic analysis as three free-range genomes were excluded due to poor quality reads (Supplementary Table S1).

### Antimicrobial Susceptibility

**Figure 1:**
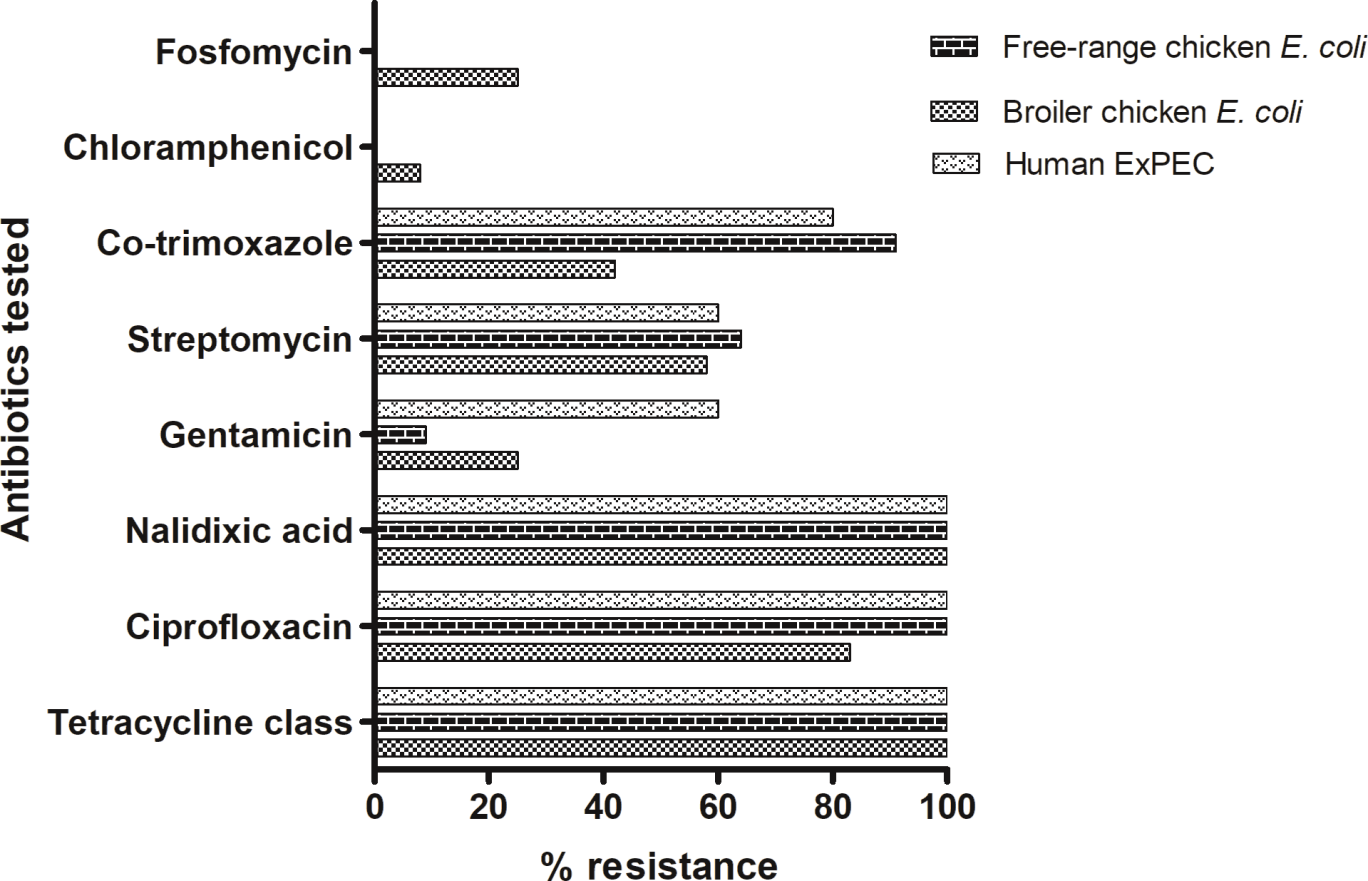
Antibiotic resistance pattern of 28 ESBL-producing *E. coli* isolates: tetracycline class includes tetracycline, oxytetracycline and chlortetracycline antibiotics. In addition, colistin antibiotic was also tested for which all isolates were found to be susceptible. Out of the 28 ESBL-producing *E. coli*, 22 (78.5%) were detected to be of MDR phenotype.

A total of 28 ESBL-producing *E. coli* isolates were screened for their antimicrobial susceptibilities to 11 antibiotics. Disc diffusion-based antimicrobial susceptibility of 11 antibiotics showed high levels of resistance among all tested *E. coli* isolates irrespective of their host origins (Figure 1). The isolates representing three groups showed comparable resistance rate (100%) towards tetracycline and quinolones class of antibiotics. Free-range *E. coli* and broiler *E. coli* isolates, including the human ExPEC, did not differ significantly in resistance with respect to any of the particular antibiotics tested. However, overall resistance scores (resistance to all the 11 antibiotics) only differed marginally among the three groups of *E. coli*. However, 78.5% of the *E. coli* isolates analyzed in this study exhibited a multidrug-resistant (MDR) phenotype. Among the 28 ESBL-positive poultry *E. coli* isolates which were tested for *in vitro* conjugation, only 12 *E. coli* isolates (comprising 7 broiler chicken *E. coli*, 3 free-range chicken *E. coli*, and 2 ExPEC) successfully transferred cefotaxime resistance to the recipient strain *E. coli*-J53. The transconjugants showed resistance to one or two antibiotics (mostly to ciprofloxacin and cotrimoxazole) in addition to beta-lactams, but none of the transconjugants was found to be resistant to all the antibiotics to which their parental *E. coli* isolates were tested resistant (Supplementary Table S2).

### Whole Genome-Based Phylogeny, MLST, and Plasmid Profiles

**Figure 2:**
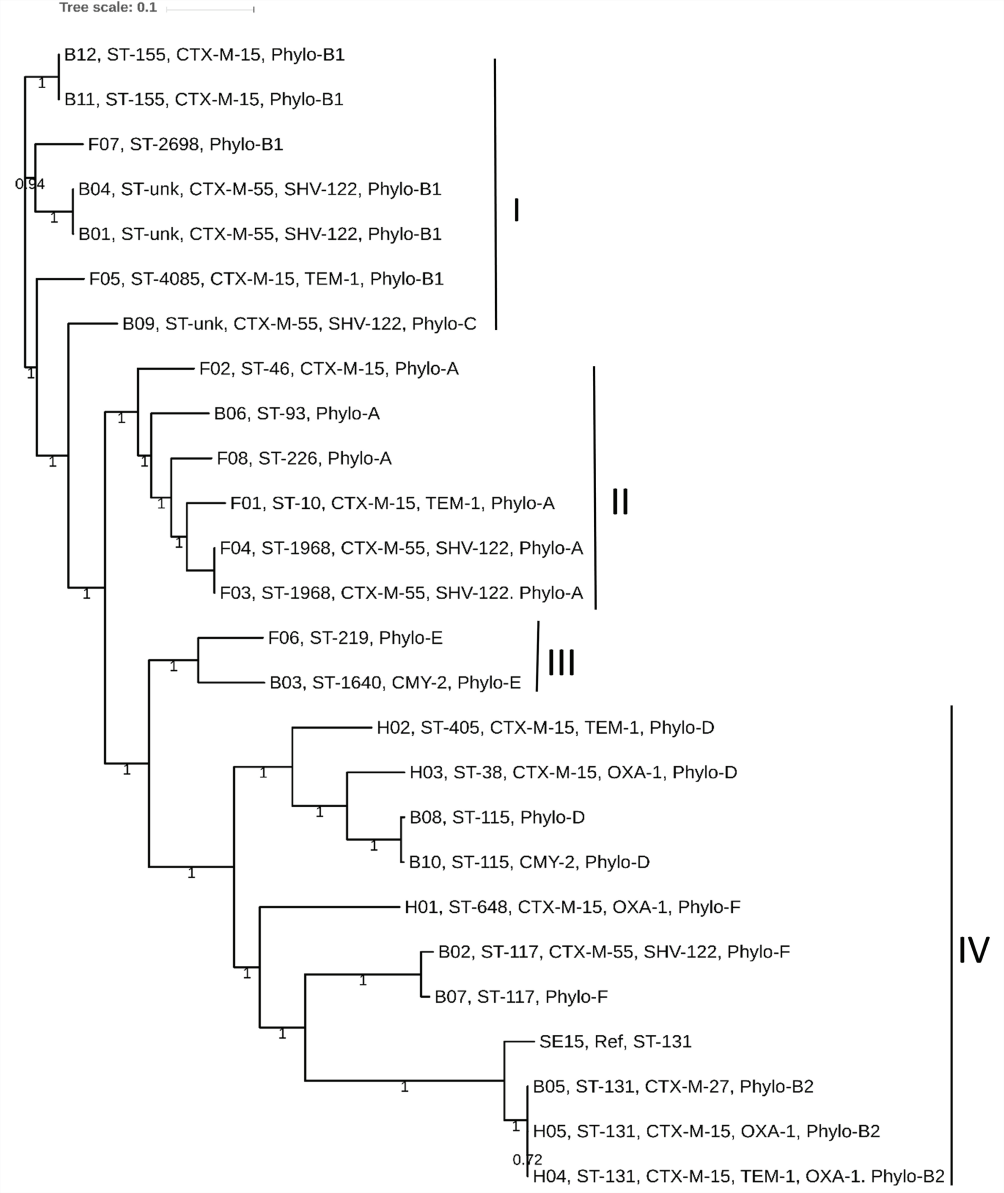
The core genome based consensus Maximum Likelihood phylogenetic tree of 25 *E. coli* genomes (12 broiler, 8 free-range and 5 human ExPEC genomes) with reference *E. coli* SE15 genome generated using Harvest. The output of Harvest was visualized using iTOL. The broiler and free-range *E. coli* isolates were present within same clusters indicating that they share similar genetic backgrounds. However, the human ExPEC isolates largely formed a distinct clade together with the genomes of broiler *E. coli* and no genome from free-range *E. coli* was represented within this clade (IV). Labeling at the tips include genome unique IDs (B01 to B12; Broiler *E. coli*, F01 to F08; Free-range *E. coli*, H01 to H05; Human ExPEC) followed by sequence types, acquired ESBL genes and the phylogroups.

We determined the phylogenetic association among 25 *E. coli* genomes obtained from broiler (12 genomes), and free-range (8 genomes) chicken samples together with 5 human ExPEC genomes. The phylogenetic tree was broadly classified into four clades: I to IV. Clades I, II, and III represented strains from both broiler and free-range chicken categories (Figure 2). However, none of the human ExPEC strains were included in these three clades. Clade I represented strains that were not clonally clustered together. Nonetheless, the presence of strains belonging to broiler and free-range chicken within clades I, II, and III indicated that the two categories of *E. coli* strains were related and perhaps shared common ancestors. The clade IV clustered all the five human ExPEC, including the reference strain SE15; this clade also clustered five other broiler chicken *E. coli* strains that are related to some of well-established ESBL associated sequence types (ST131, ST117, and ST115) and virulent phylotypes (B2, D, and F). Isolates from free-range chicken were not related to this clade. The human ExPEC strains were only related to clade IV possibly because of the small sample size and a limited number of sequence types they belonged to and that they originated from a single geographic location. None of the free-range *E. coli* belonged to B2, D, and F phylogroups that were represented by the human ExPEC genomes whereas two broiler *E. coli* isolates were belonging to phylogroup B2 and D each and one to phylogroup F. Using *in silico* MLST (Larsen et al., 2012), we identified 13 STs, in poultry *E. coli* which included globally successful sequence types such as ST117 (9%), ST131 (4.3%), and ST10 (4.3%). Using the PlasmidFinder database (Carattoli et al., 2014), we identified a total of 14 different plasmid replicons (including FIA, FIB, FIC, FII, B/O/K/Z, Col, I1, I2, I7, N, po111, Q1, R, and Y) to be present in our collection of *E. coli* isolates (Supplementary Table S1). FIB (73%) and FII (73%), were the most common plasmid replicons identified among the poultry and human ExPEC genomes analyzed (Supplementary Table S1).

### Whole Genome Sequence-Based Identification of Antibiotic Resistance and Virulence Genes

**Figure 3:**
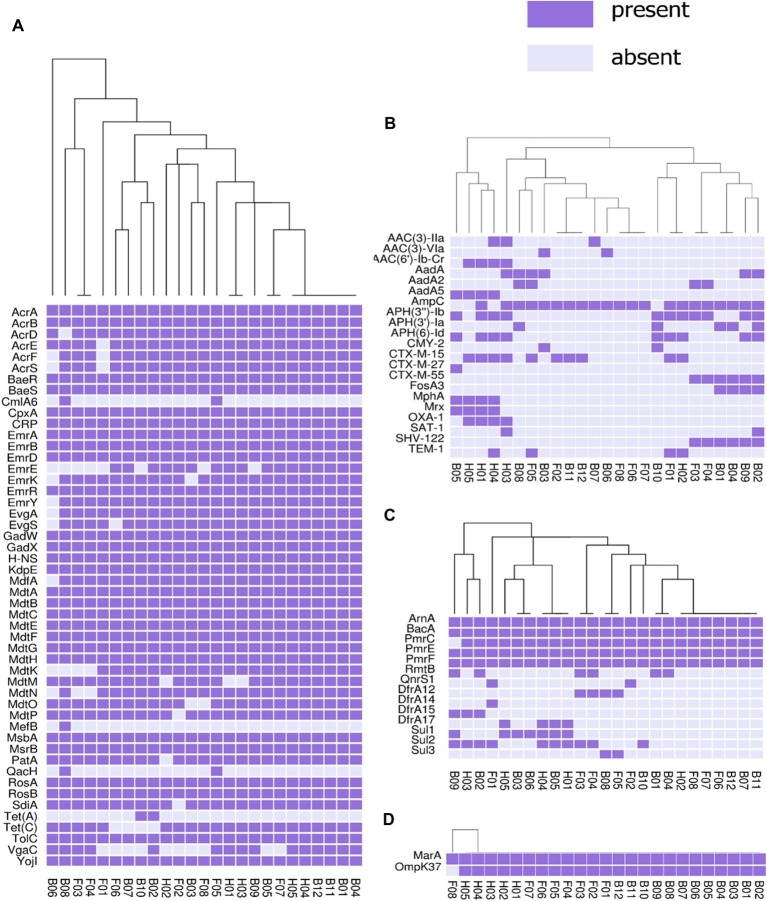
Cluster heat map of 25 *E. coli* genomes (12 broiler, 8 free-range and 5 human ExPEC genomes) showing the presence and absence of 92 antibiotic resistance genes belonging to different categories **(A)** antibiotic efflux, **(B)** antibiotic inactivation, **(C)** target alteration, protection and replacement, **(D)** reduced permeability to antibiotics. Results of resistance clustering indicated that the three groups of *E. coli* isolates including broiler, free-range and human isolates represented clusters with mixed strains which shared resistance gene absence/ presence profile with each other. Gene names are represented on Y axis and the *E. coli* genomes are listed on the X axis (B01 to B12; Broiler *E. coli*, F01 to F08; Free-range *E. coli*, H01 to H05; Human ExPEC). Detail description of genomes can be found in Supplementary Table S1.

**Figure 4:**
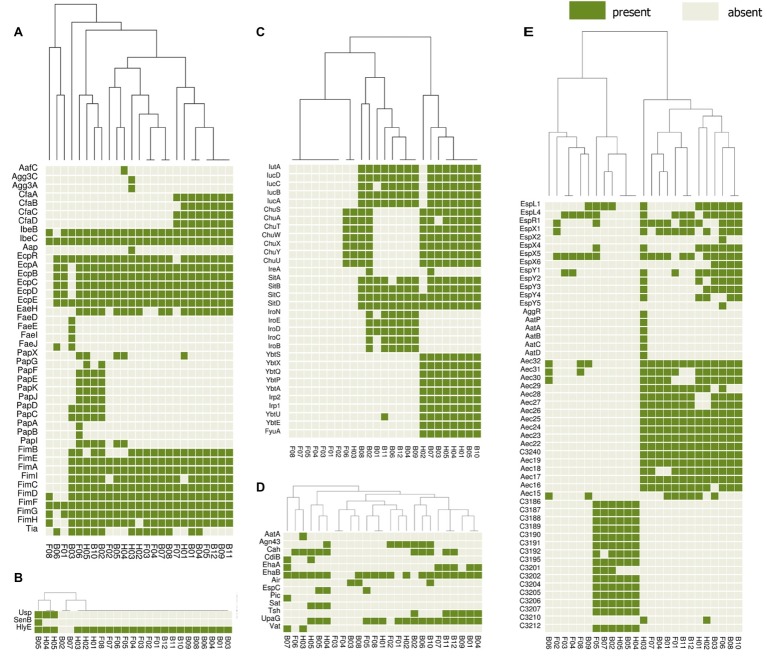
Cluster heat map of 25 *E. coli* genomes (12 broiler, 8 free-range and 5 human ExPEC genomes) showing the presence and absence of 143 virulence genes belonging to different categories **(A)** adherence, **(B)** toxins, **(C)** iron uptake, **(D)** auto-transporters, **(E)** secretion systems. The virulence based clustering indicated that the three groups of *E. coli* including broiler, free-range and human ExPEC shared virulence gene profiles with each other. Gene names are represented on Y axis and the *E. coli* genomes are listed on the X axis (B01 to B12; Broiler *E. coli*, F01 to F08; Free-range *E. coli*, H01 to H05; Human ExPEC). Detail description of genomes can be found in Supplementary Table S1.

The presence or absence of different categories of virulence and antimicrobial resistance genes in each of the isolates are displayed in Figures 3, 4. The heat maps indicate the status of antimicrobial resistance and virulence genes and the dendrogram discerns the relationships among the isolates based on the presence/absence profiles of the genes analyzed.

In this analysis (Figure 3), 92 out of 2,678 different resistance genes were detected among 25 *E. coli* isolates comprising 12 broiler chicken, 8 free-range chicken, and 5 human ExPEC strains. The three groups of *E. coli* isolates did not demonstrate distinct resistance gene profiles (absence/presence) compared to each other. On the other hand, many strains belonging to the three groups shared resistance gene profiles as evident from the dendrogram. However, when the aggregate resistance gene scores were analyzed, free-range chicken *E. coli* isolates exhibited significantly lower (*p* = 0.033) resistance score (median = 54.5) compared to human ExPEC isolates (median = 63) while the resistance gene scores were statistically comparable (*p* = 0.059) between broiler chicken (median = 56) and human ExPEC isolates (median = 63). Poultry-derived ESBL-producing *E. coli* isolates in this study harbored different variants of ESBL genes including the *bla*_CTX-M-15_ (40%), *bla*_CTX-M-27_ (4%), *bla*_CTX-M-55_ (24%), *bla*_TEM-1_ (16%), and *bla*_SHV-122_ (24%) (Figure 3). However, common among them was *bla*_CTX-M-15_, which was present in four clonally diverse STs, including ST10, ST46, ST155, and ST4985. The correlation between resistance genotypes (presence of resistance genes) and phenotypes (resistant phenotype) was high for tetracycline (74% agreement), followed by aminoglycosides (65% agreement), sulfonamides (53% agreement), and phenicols (50% agreement) whereas only 6 out of 21 isolates resistant to fluoroquinolone harbored *aac (6)-lb-cr* (4 isolates) and *QnrS*1 (2 isolates) genes.

In addition, a second heat map was constructed to screen major groups of known virulence genes across the whole genome sequenced *E. coli* strains (Figure 4). This analysis revealed presence of 143 out of 2,681 different virulence genes analyzed among the set of 25 *E. coli* isolates. The heat map demonstrated no unique virulence gene profile for a particular *E. coli* group (broiler or free-range chicken isolates and human ExPEC), as there was no cluster within the heat map that included only individual strains from the same group. This indicates that strains from all three groups shared virulence gene profiles except for iron acquisition systems as free-range *E. coli* harbored very few iron acquisition genes. Similar to the resistance scores, the *E. coli* isolates from free-range chicken showed significantly lower (*p* = 0.008) aggregate virulence gene scores (median = 27) compared to human ExPEC isolates (median = 68) in contrast to *E. coli* from broiler chicken (median = 62), which showed statistically comparable values (*p* = 0.075) with human ExPEC isolates. Similarly, when presence of 15 ExPEC associated genes (*pap* A/C/E/F/G, *fim*H, *pic*, *sat*, *tsh*, *vat*, *iut*A, *ire*A, *iro*N, *fyu*A, and *usp*) were analyzed (Pitout, 2012), least number of ExPEC-associated genes (median and range) were detected in free-range *E. coli* isolates (1, 0–5) whereas, the broiler and human ExPEC isolates were comparable with median and range of 4, 0–9 and 3, 1–9, respectively.

### Virulence-Associated Phenotypes of Extended-Spectrum β-Lactamase-Producing *E. coli* Isolates

#### Biofilm Formation

**Figure 5:**
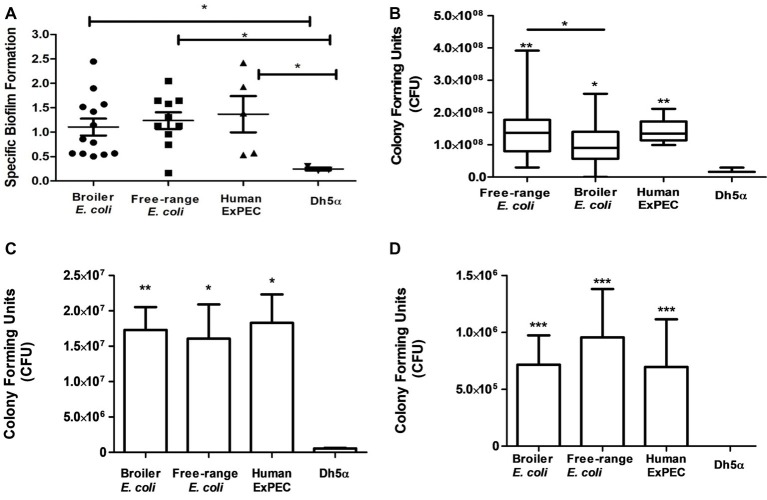
*In vitro* functional characterization of 11 free-range chicken *E. coli* isolates, 12 broiler chicken *E. coli* isolates and 5 human ExPEC including the control *E. coli* DH5α. **(A)** Biofilm formation capabilities determined in M-63 minimal media using 96-well microtiter plate method. **(B)** Resistance to serum bactericidal activity against 50% human serum. **(C)** Adhesion and **(D)** invasion capabilities on T24 bladder epithelial cells. All 28 *E. coli* strains demonstrated significant correlation with the above tested virulence phenotypes compared to the negative control strain, *E. coli* DH5α. Experiments were performed twice (serum assay)/thrice (adhesion, invasion, and biofilm formation assays) in triplicates and statistical values were obtained using the nonparametric Mann-Whitney U test. 5.01. *p < 0.05; **p < 0.01; ***p < 0.001.

Biofilms were produced by all the identified 28 *E. coli* isolates corresponding to 11 free-range, 12 broiler and 5 human ExPEC isolates (Figure 5A), as compared to the negative control DH5α. Moreover, biofilm formation capability was found to be similarly prevalent among free-range *E. coli* and broiler isolates (*p* = >0.05) which indicates their similar propensities to form biofilms. However, free-range *E. coli* isolates produced optically denser biofilms in comparison to broiler *E. coli* isolates. Overall, the biofilm formation capabilities of both categories of poultry *E. coli* (free-range and broiler) isolates were comparable to the biofilm formation capabilities of human extraintestinal pathogenic *E. coli* (ExPEC) isolates.

#### Serum Resistance Assay

All identified *E. coli* (*n* = 28) strains were tested for their capacity to resist the serum bactericidal activity when incubated with 50% human serum. Strains that survived from the bactericidal activity of the serum even after 3 h of incubation were defined as serum resistant and those that did not survive or increase their CFU counts were considered serum sensitive. Interestingly, all the 28 *E. coli* strains, irrespective of free-range, broiler, or human clinical origins, were resistant to human serum as compared to the negative control strain, *E. coli* DH5α (Figure 5B). Although the broiler group of *E. coli* demonstrated serum resistance, the mean CFU count of this group was significantly less than free-range and human *E. coli* groups (*p* = 0.049 and *p* = 0.008, respectively). However, no significant difference (*p* = 0.738) was observed between the mean serum resistance of free-range chicken and human *E. coli* groups.

#### Adhesion Property

The *E. coli* isolates (*n* = 28) were also tested for their adherence to the human T24 bladder epithelial cells. Free-range and broiler *E. coli* strains demonstrated significant adherence to T24 cell line in comparison to DH5α strain (Figure 5C). The adhesion capabilities of the poultry *E. coli* strains (free-range and broiler) were comparable to the adhesion capabilities of human ExPEC strains and lacked any significant differences (*p* > 0.05). However, the broiler chicken *E. coli* strains were slightly more (mean 1.73 × 10^7^ CFU) adherent to T24 cells rather than the free-range chicken *E. coli* isolates (mean 1.6 × 10^7^) but statistically these deviations were non-significant. The mean CFU counts entailing the adherence of human strains to T24 cells were 1.83 × 10^7^.

#### Invasion Capability

All those strains included in the adhesion assay were also tested for their invasion property in T24 cells by gentamicin (100 μg/ml) protection assay (Figure 5D). Both groups of poultry strains (broiler and free-range chicken) demonstrated prominent invasion into T24 cell line compared to the invasion capabilities of the human ExPEC strains. In contrast to the adhesion capability, the group of free-range chicken *E. coli* strains showed slightly more invasion rates than the group of broiler chicken *E. coli* strains (mean 7.1 × 10^6^ CFU) but the two groups did not differ significantly. The mean invasion capability of free-range chicken *E. coli* in T24 cells was comparable to the mean invasion capability of the human ExPEC strains with 9.5 × 10^6^ CFU and 6.9 × 10^6^ CFU, respectively as the difference was found to be statistically non-significant (*p* = 0.382).

## Discussion

The prevalence of virulence potential in ESBL-producing *E. coli* originating from healthy production chickens could be a significant factor for both human and animal health. In this baseline study, we tested ESBL-producing *E. coli* isolates from healthy chickens (broiler and free-range) with regard to their phenotypic virulence traits and extensive antimicrobial resistance. This *in vitro* virulence and resistance testing revealed that the ESBL-producing *E. coli* in poultry demonstrated strong virulence and resistance attributes, irrespective of their host origins such as broiler or free-range chicken. *E. coli* is known to be a highly diverse species that is frequently associated with antimicrobial resistance. Here, we carried out whole-genome sequencing of *E. coli* isolates obtained from different regions of India, about which such data are largely lacking. We then determined the genomic diversity and/or similarity with respect to core genome phylogeny, STs, virulence and resistance gene profiles. Our observations revealed that *E. coli* in sampled settings possess high diversity at the level of phylogeny and sequence types.

In our study, about 78.5% of the ESBL-producing *E. coli* were detected to be of multi-drug resistant phenotype. They were frequently resistant to most of the antibiotics tested such as tetracyclines, fluoroquinolones, aminoglycosides, and sulfonamides. This coincides with the use of tetracyclines (oxytetracycline and chlortetracycline), fluoroquinolones (ciprofloxacin and enrofloxacin), aminoglycosides (gentamicin and streptomycin), and sulfonamides (co-trimoxazole) in meat production industry in India (Laxminarayan and Chaudhury, 2016; Brower et al., 2017; Kakkar et al., 2017). The common ESBL genotype identified was the *bla*_CTX-M-15_, which was present in four clonally diverse STs namely, ST10, ST46, ST155, and ST4985. Presence of *bla*_CTX-M-15_ in diverse STs has also been recently reported (Mshana et al., 2016; Musicha et al., 2017) and globally, the *bla*_CTX-M-15_ gene was reported to be strongly linked with ST131 *E. coli* lineage (Price et al., 2013). It has been already evident that *bla*_CTX-M-15_ can spread by genetic mobile elements such as conjugative plasmids (Inwezerua et al., 2014; Irrgang et al., 2017). Further studies are required in India to accurately estimate the prevalence of *bla*_CTX-M-15_ and other variants and correlate its association with different successful lineages causing multi-drug resistant infections. Conjugation experiments suggest that the ESBL production in poultry *E. coli* isolates may be associated with conjugative plasmids as also reported by others, that *E. coli* isolates of poultry origin harbor conjugative plasmids (Johnson et al., 2012). Sequence types 155, 115, and 117 constitute the most clonal clades in the population structure of our collection. Interestingly, corroborating with the virulence and resistance gene profiling results, the phylogeny results also revealed that the broiler or free-range chicken *E. coli* did not form two homogenous groups, instead, they demonstrated mixed clustering. These findings suggest that irrespective of the host origin the *E. coli* from broiler or free-range chicken share genetic backgrounds with each other and may belong to the same ancestral lineage(s) that could be the natural inhabitants of the poultry. The presence of MDR-bacteria in food sources such as poultry is a matter of public health concern as these MDR bacteria could potentially transfer their resistant traits to food-borne pathogens. Moreover, the presence of MDR-*E. coli* in the intestines of slaughtered birds can contaminate the carcasses during slaughter and processing, and thereby spread to humans directly or *via* the food chain.

We then analyzed the ESBL-producing poultry *E. coli* isolates to characterize the virulence potential of these isolates. Certain *in vitro* phenotypic traits such as biofilm formation, serum resistance, adhesion and invasion properties may correspond with or give rise to, the pathogenic potential of the *E. coli* isolates (Nandanwar et al., 2014; Mitchell et al., 2015).

Bacteria produce biofilm in order to protect themselves from host defense elements, antibiotics, and detergents; biofilms allow them to be in close proximity to other pathogens, so as to horizontally acquire genetic material and become either more antibiotic resistant or virulent (Naves et al., 2008). In this study, 100% of tested *E. coli* strains demonstrated biofilm formation ability and the range of biofilm formation did not vary between the two groups of chicken *E. coli* isolates (broiler and free-range chicken). Thus, we confirm that *E. coli* isolates of food origin are being able to survive on polystyrene 96-well microtiter plates. This is an important observation as these *E. coli* can survive on the food-processing surfaces and potentially persist there withstanding the unfavorable conditions and re-contaminate food products. This is supported by a previous report on *E. coli* isolates obtained from a poultry slaughterhouse (Rodrigues et al., 2010).

To gain further understanding of the ability of poultry *E. coli* isolates to cause infections in humans, the 23 poultry *E. coli* isolates along with 5 human ExPEC isolates were assessed for their ability to attach and invade mammalian T24 bladder epithelial cells *in vitro.* All 23 poultry *E. coli* showed stronger adhesion and invasion capabilities comparable to the human ExPEC isolates. Interestingly, the free-range *E. coli* isolates demonstrated higher invasion strength than the broiler *E. coli* isolates. In this study, virulence factors, including *fim*, *pap*, *tsh*, *upa,* and *ibeB*, were frequently observed (Figure 4) that are reported to be associated with virulence properties (Rodriguez-Siek et al., 2005; Antão et al., 2009; Wang et al., 2012). Effective cellular adhesion followed by invasion are the key events in pathogenicity; the above observation reaffirms the potential of healthy poultry derived *E. coli* to be of concern. Moreover, the poultry *E. coli* could use similar mechanisms to strongly adhere to chicken meat (LeStrange et al., 2017), and therefore this could be a potential method of foodborne transmission of pathogenic and or MDR *E. coli* to humans.

Resistance to the serum bactericidal activity is an important virulence determinant in *E. coli* strains where it contributes to pathogenesis particularly in extraintestinal infections (Mellata et al., 2003). A strong correlation between serum resistance and the ability of poultry *E. coli* to infect the internal organs of chicken was reported previously (Mellata et al., 2003). Here, we detected serum resistance for all poultry *E. coli* isolates including that of human ExPEC isolates. The free-range *E. coli* isolates exhibited significantly higher serum resistance than the broiler *E. coli*. In order to survive in extraintestinal sites, microbes, on the one hand, have to overcome harsh environments and on the other hand, have to acquire micronutrients such as iron. Consequently, the poultry *E. coli* isolates possessed several siderophore genes such as *iroN*, *chuA,* and *sitD*. However, the free-range *E. coli* isolates harbored very few siderophore genes compared to the broiler *E. coli* isolates, the reason for such an observation could be found in another study which reported that environmental *E. coli* isolates are less likely to contain genes associated with aerobactin and yersiniabactin (Searle et al., 2015). The country chicken could have mostly acquired these bacteria from the environment as they feed by free-ranging. All these isolates analyzed in this study were ESBL producers and most of them harbored multiple plasmid types. It is reported that the presence of certain ESBL-plasmids could contribute to competitive fitness and serum survival of the *E. coli* strains (Ranjan et al., 2018). Plasmid replicon results as reported in this study suggest that this may be the case as we observed the presence of multiple plasmids that have been linked with both resistance and virulence, the most predominant being FIB (73%) and FII (73%) (Johnson et al., 2007).

In conclusion, this baseline study has provided insights into resistance and virulence–related genotypes and phenotypes of ESBL-producing *E. coli* from broiler and free-range chicken in India. Also, it has revealed a high diversity of lineages of ESBL-producing *E. coli* circulating in India. Although the human ExPEC isolates could be largely separated from the poultry *E. coli* isolates when virulence-associated phenotypes were taken into account, the substantial overlap was still evident. Particularly, the broiler chicken *E. coli* and human ExPEC isolates harbored large number of virulence and resistance genes compared to free-range chicken *E. coli*. This suggests that the transfer of ESBL-producing isolates from contaminated meat may likely contribute to the colonization of drug-resistant bacteria in humans through consumption of broiler chicken products that are not properly cooked and that certain *E. coli* clones from broiler-poultry may indeed have the potential to cause infection in humans. Further work is required to ascertain the genomic similarity among large number of geographically matched contemporaneous ESBL-producing *E. coli* isolates from humans and retail chicken meat. This work significantly contributes to the understanding of the epidemiology of ESBL-producing *E. coli* particularly from animal foods such as poultry.

## Data Availability Statement

The datasets generated for this study can be found in the GenBank accession numbers of the 10 genomes sequenced in this study are RDBX00000000 (NAEC1028), RDBY00000000 (NAEC1042), RDBZ00000000 (NAEC1115), RDCA00000000 (NAEC1132), RDCB00000000 (NAEC1134), RDCC00000000 (NAEC1135), RDCD00000000 (NAEC1136), RDCE00000000 (NAEC1146), RDCF00000000 (NAEC1147), and RDCG00000000 (NAEC1154).

## Author Contributions

DC and NA provided overarching supervision to the study. AH designed and conducted the study. AR helped in performing virulence assays. SS, AS, and TS helped in analyzing genome sequence data. MA, HW, LW, and NS helped in preparation of the manuscript.

### Conflict of Interest

The authors declare that the research was conducted in the absence of any commercial or financial relationships that could be construed as a potential conflict of interest.

## Footnotes

^1^http://www.genomicepidemiology.org/
