# Supplementary material for: Genomic and Functional Characterization of Poultry Escherichia coli From India Revealed Diverse Extended-Spectrum β-Lactamase-Producing Lineages With Shared Virulence Profiles
Source: Front Microbiol. 2019 Dec 3;10:2766. doi: 10.3389/fmicb.2019.02766 (PMC6901389; doi:10.3389/fmicb.2019.02766)
Supplement: Supplementary file 1 [file Table_1.DOCX]

Table S1: List of strains employed in this study and their meta-data.

| **S. No.** | **Strain** | **Host/Source** | **Location (State)** | **Accession No.** | **Unique ID for *in silico* analysis** | **MLST** | **Plasmid profiles** |
| --- | --- | --- | --- | --- | --- | --- | --- |
| 1 | **NA1001** | B-C | T | MKWV00000000 | B01 | UNK | FII, I7, FIB, I2, FIC |
| 2 | **NA1002** | B-C | T | MKWW00000000 | B02 | ST 117 | FII, FIC, FIB, N |
| 3 | **NA1003** | B-C | T | MKWX00000000 | B03 | ST 1640 | FIB, FII, B/O/K/Z |
| 4 | **NA1004** | B-C | T | MKWY00000000 | B04 | UNK | FIC, I1, FIB, N, I2 |
| 5 | **NAEC1** | B-M | T | MLAZ00000000 | B05 | ST 131 | FIB, FIA, FII, Col |
| 6 | **NAEC2** | B-C | T | MLBA00000000 | B08 | ST 115 | I1, I2 |
| 7 | **NAEC3** | B-M | K | MLBB00000000 | B09 | UNK | FIC, FIB, I1, N, Col, FII, Q1 |
| 8 | **NAEC4** | B-M | K | MLBC00000000 | B10 | ST 115 | FII, B/O/K/Z, FIB, N |
| 9 | **NAEC5** | B-C | AP | MLBD00000000 | B11 | ST 155 | Col, FIB, N |
| 10 | **NAEC6** | B-C | AP | MKWZ00000000 | B12 | ST 155 | FIC, Y, FIB |
| 11 | **NAEC**  **1028*** | B-C | T | RDBX00000000 | B06 | ST 93 | FIB, FII, Col |
| 12 | **NAEC**  **1042*** | B-M | T | RDBY00000000 | B07 | ST 117 | FIB, FII, Col |
| 13 | **NAEC**  **1115*** | F-C | K | RDBZ00000000 | F01 | ST 10 | NPF |
| 14 | **NAEC**  **1123*** | F-M | K | ND | ND | ND | ND |
| 15 | **NAEC**  **1132*** | F-C | K | RDCA00000000 | F02 | ST 46 | NPF |
| 16 | **NAEC**  **1134*** | F-C | K | RDCB00000000 | F03 | ST 1968 | FIB, FIC, N, Pol111, FII |
| 17 | **NAEC**  **1135*** | F-C | K | RDCC00000000 | F04 | UNK | FIB, FIC, N, Pol111, FII |
| 18 | **NAEC**  **1136*** | F-C | AP | RDCD00000000 | F05 | ST 4985 | R |
| 19 | **NAEC**  **1145*** | F-C | AP | ND | ND | ND | ND |
| 20 | **NAEC**  **1146*** | F-M | T | RDCE00000000 | F06 | ST 219 | FII |
| 21 | **NAEC**  **1147*** | F-M | T | RDCF00000000 | F07 | ST 2698 | FII |
| 22 | **NAEC**  **1154*** | F-M | T | RDCG00000000 | F08 | ST 226 | NPF |
| 23 | **NAEC**  **1148*** | F-M | AP | ND | ND | ND | ND |
| 24 | **NA023** | U | M | JSXK00000000 | H01 | ST 648 | FIB, FIA, FII |
| 25 | **NA081** | U | M | JSXM00000000 | H02 | ST 405 | FIB, Y, FII |
| 26 | **NA090** | U | M | MVIO00000000 | H03 | ST 38 | FIB, FII |
| 27 | **NA097** | U | M | JSXJ01000000 | H04 | ST 131 | FII, FIA |
| 28 | **NA114** | U | M | MIPU01000000 | H05 | ST 131 | FII, FIA |

* Newly sequenced genomes in this study

Location (state): T= Telangana, K= Karnataka, AP= Andhra Pradesh, M= Maharashtra

Host/source: **B-C**= Broiler, ceca. **B-M**= Broiler, raw meat. **F-C**= Free-range, ceca.

**F-M**= free-range, raw meat. **U=** Urine

ND = Genomes were excluded from molecular analysis due to poor quality reads

UNK = Sequence type unknown

NPF = Plasmid not found

Table S2: Results of antimicrobial susceptibilities of transconjugants in comparison with their parental strains

| **S. No.** | **Strains** | **Host origin (chicken)** | **^a^Parental strains^R^** | **^a^Transconjugants^R^** | **Replicon Type in Transconjugants** |
| --- | --- | --- | --- | --- | --- |
| 1 | NA1001 | Broiler | Tet, Cip, Hlg | Cip | FII |
| 2 | NA1004 | Broiler | Tet, Cip, Hlg | Cip | N |
| 3 | NAEC1 | Broiler | Tet, Cot, Cip |  | FIB |
| 4 | NA1042 | Broiler | Cip, Hlg |  | ND* |
| 5 | NAEC5 | Broiler | Tet, Cip | Cip | N |
| 6 | NAEC6 | Broiler | Tet, Cip | Cip | FIB |
| 7 | NAEC3 | Broiler | Tet, Cot, Cip, Hlg, Fos | Cip, Cot | N |
| 8 | NA1134 | Free-range | Tet, Cot, Cip, Hlg | Cip, Cot | N |
| 9 | NA1135 | Free-range | Tet, Cot, Cip, Hlg | Hlg | N |
| 10 | NA1136 | Free-range | Tet, Cot, Cip, Hlg | Cip, Cot | ND* |
| 11 | NA23 | Human ExPEC | Tet, Cot, Cip, Hlg | Tet, Cot | FIA |
| 12 | NA97 | Human ExpEC | Tet, Cot, Cip, Hlg | Cip, Cot | FIA |

^a^ Parental strains and transconjugants were tested and compared for antimicrobial susceptibilities against a total of 6 antibiotics; tetracycline (Tet), co-trimoxazole (Cot), ciprofloxacin (Cip), gentamicin (Hlg), chloramphenicol (C) and fosfomycin (Fos).

^R^ Antibiotics to which the strains were resistant are mentioned in the column

*ND = Not detected

Table S3: Genome features and assembly statistics of 10 whole genome sequenced poultry *E. coli* isolates

| **S. No.** | **Strain Name** | **Number of contigs**  **>=500bp** | **Genome size (bp)** | **G+C content**  **(%)** | **No. of CDS** | **Coding %** | **No. of rRNAs** | **No. of tRNA** |
| --- | --- | --- | --- | --- | --- | --- | --- | --- |
| 1 | NAEC1154 | 116 | 4613853 | 50.79 | 4436 | 87.2 | 10 | 64 |
| 2 | NAEC1147 | 120 | 4938369 | 50.7 | 4867 | 87.6 | 10 | 70 |
| 3 | NAEC1146 | 166 | 5082262 | 50.53 | 4829 | 87.4 | 13 | 66 |
| 4 | NAEC1136 | 57 | 4889074 | 50.76 | 4663 | 88 | 10 | 67 |
| 5 | NAEC1135 | 173 | 4845393 | 50.71 | 4781 | 86.9 | 6 | 64 |
| 6 | NAEC1134 | 172 | 4850227 | 50.72 | 4780 | 86.9 | 9 | 68 |
| 7 | NAEC1132 | 123 | 4507061 | 50.85 | 4353 | 87.5 | 7 | 61 |
| 8 | NAEC1115 | 112 | 4670108 | 50.86 | 4490 | 87.7 | 8 | 58 |
| 9 | NAEC1042 | 206 | 5105095 | 50.81 | 4964 | 86.5 | 6 | 61 |
| 10 | NAEC1028 | 256 | 4894553 | 51.04 | 4781 | 86.1 | 8 | 47 |
